# Metagenomic analysis suggests broad metabolic potential in extracellular symbionts of the bivalve *Thyasira* cf. *gouldi*

**DOI:** 10.1186/s42523-020-00025-9

**Published:** 2020-03-05

**Authors:** Bonita McCuaig, Lourdes Peña-Castillo, Suzanne C. Dufour

**Affiliations:** 1 Department of Biology, Memorial University of Newfoundland, St. John’s, NL Canada; 2 Department of Computer Science, Memorial University of Newfoundland, St. John’s, NL Canada

**Keywords:** Sulfur-oxidizing, Thyasiridae, Magnetotactic, Facultative, Mollusc, Mixotrophic

## Abstract

**Background:**

Next-generation sequencing has opened new avenues for studying metabolic capabilities of bacteria that cannot be cultured. Here, we provide a metagenomic description of chemoautotrophic gammaproteobacterial symbionts associated with *Thyasira* cf. *gouldi*, a sediment-dwelling bivalve from the family Thyasiridae. Thyasirid symbionts differ from those of other bivalves by being extracellular, and recent work suggests that they are capable of living freely in the environment.

**Results:**

*Thyasira* cf. *gouldi* symbionts appear to form mixed, non-clonal populations in the host, show no signs of genomic reduction and contain many genes that would only be useful outside the host, including flagellar and chemotaxis genes. The thyasirid symbionts may be capable of sulfur oxidation via both the sulfur oxidation and reverse dissimilatory sulfate reduction pathways, as observed in other bivalve symbionts. In addition, genes for hydrogen oxidation and dissimilatory nitrate reduction were found, suggesting varied metabolic capabilities under a range of redox conditions. The genes of the tricarboxylic acid cycle are also present, along with membrane bound sugar importer channels, suggesting that the bacteria may be mixotrophic.

**Conclusions:**

In this study, we have generated the first thyasirid symbiont genomic resources. In *Thyasira* cf. *gouldi*, symbiont populations appear non-clonal and encode genes for a plethora of metabolic capabilities; future work should examine whether symbiont heterogeneity and metabolic breadth, which have been shown in some intracellular chemosymbionts, are signatures of extracellular chemosymbionts in bivalves.

## Background

Many species of marine bivalves living near oxic-anoxic boundaries form nutritional symbioses with chemoautotrophic bacteria, which are maintained in or on the host’s gills [1–4]. In such associations, called chemosynthetic symbioses or chemosymbioses, the bacteria provide the host with nutrients and protection from chemical stress, while the host constitutes a protective and suitable environment for the bacterial symbionts [2, 5, 6]. The metabolism of symbionts allows hosts to colonize new and often nutrient-poor niches and contributes to their ecological and evolutionary success, as moving into a niche with less competition for resources can lead to evolutionary radiation [7, 8].

Symbionts can be acquired by new generations of hosts in various ways. Vertical transmission is the transfer of bacteria from one generation to the next through gametes, most commonly the eggs. In horizontal transmission, hosts are inoculated by symbionts released by nearby adults, whereas in environmental transmission, juveniles are inoculated from a free-living symbiont population [3, 6]. While the latter mode of transmission does not guarantee symbiont transfer, it does confer some advantages to both partners. Bacteria that maintain a free-living population can avoid genomic reduction, which, in some vertically transmitted bacteria, can result in the deletion of genes that no longer improve their fitness. Once genomic reduction occurs (via vertical transmission), some symbionts may not be able to survive without their host, and become reliant upon them [9]; this does not occur in symbionts that maintain a functional environmental population. The maintenance of variation within free-living bacterial populations can also benefit the host, which can be inoculated by symbionts that are well adapted for the local environment, and not necessarily the strain present in their progenitors. Environmentally acquired symbionts could potentially form diverse or heterogeneous populations within host individuals if multiple symbionts are acquired from a genetically diverse founding population.

Symbionts can supplement nutrients that are lacking in the host’s diet, or simply provide an additional source of nutrients. The mode of nutrient transfer from symbiont to host varies by relationship, and in many cases, is not well defined. Some symbionts have been shown to actively transfer nutrients to their host, others have “leaky membranes” that allow nutrients to escape the bacteria, and in other cases the host consumes the bacteria through phagocytosis [10, 11]. Some metabolic cycles of the symbionts may remove toxins present in the environment, providing the host protection from these compounds [12, 13]. In chemoautotrophic symbionts, the sulfur oxidation (sox) and dissimilatory sulfate reduction (dsr) based sulfur metabolism may remove toxic sulfur compounds while providing energy for carbon fixation. The nitrite reduction (nir) pathway removes harmful nitrogen compounds by using them as an electron sink, but this process is not always coupled with carbon fixation [14]. One approach to examining the metabolic potential of chemoautotrophic symbionts is to perform genomic, or metagenomic sequencing [15–18]. By identifying key genes in sequencing data, the metabolic capabilities of symbionts can be inferred.

The bivalve genus *Thyasira* (Family Thyasiridae) contains both symbiotic and asymbiotic species, a seemingly unique condition among bivalve genera [19, 20]. In contrast to other clams, thyasirids maintain their symbionts among the microvilli of gill epithelial cells, as described in some mussels; such extracellular symbioses have been considered more primitive than intracellular symbioses [6, 21–23]. Chemosymbiotic thyasirids are mixotrophs that appear to rely on particulate food to a greater extent when symbiont abundance is low [24], or at times when environmental sulfide concentrations are low [25]. All thyasirid symbionts identified to date are gammaproteobacteria [23, 25, 26]. The thyasirid symbionts are clustered into divergent groups which include both symbiotic and free-living sulfur-oxidizing bacteria [23]. Enzymatic and PCR techniques have shown the presence of ribulosebisphosphate carboxylase (RuBisCO) and adenylylsulphate reductase in the symbionts of all chemosymbiotic thyasirids investigated [23, 25].

In Bonne Bay, Newfoundland, Canada, gammaproteobacteria have been found living extracellularly on the gills of thyasirid clams identified as *Thyasira* cf. *gouldi* OTUs 1 and 2 [20]. The symbionts are found in high abundance among the elongate microvilli of abfrontally expanded gill filaments [20]. This relationship has been observed over multiple years in three sampling sites, and all specimens of *T*. cf. *gouldi* OTUs 1 and 2 whose gills have been examined using thin sectioning and transmission electron microscopy (i.e., over 200 specimens) harboured large populations of visibly homogenous bacterial symbionts [20, 24, 27, 28]. Phylogenetic analysis using 16S rRNA gene sequences have identified three distinct symbiont phylotypes (A – C) hosted by the two symbiotic *T.* cf. *gouldi* OTUs [27, 28]. There was no apparent co-speciation between host and symbiont as both clam OTUs could host any one of the three symbiont phylotypes, and there is evidence of multiple RuBisCO types in a single host [27, 28]. The three bacterial 16S rRNA gene phylotypes cluster during phylogenic analysis and are closely related to the *Thyasira flexuosa* symbiont and to tubeworm symbionts (notably those associated with *Riftia pachyptila*) as well as free-living sulfur oxidizing bacteria [27, 28]. Stable isotope analysis of *T.* cf. *gouldi* supports chemoautotrophic activity and nutrient transfer from bacteria to host, notably through a lower δ^15^N value than in non-symbiotic, co-occurring bivalves (representing a greater contribution of nutrients from a low trophic level, likely through chemoautotrophic primary production); tissue δ^13^C values were less negative than in other chemosymbiotic thyasirids, as expected due to the presence of RuBisCO type II [29]. Taken together, the consistent presence of abundant gill-associated sulfur-oxidizing bacteria in numerous *T*. cf. *gouldi* individuals and the stable isotope data are strongly indicative of a symbiotic relationship rather than an environmental contamination. *T.* cf. *gouldi* symbionts have been identified within surrounding sediment samples, supporting an environmental mode of transmission and the existence of a free-living symbiont population [30].

We present here the first genomic analysis of a thyasirid symbiont, that of *T.* cf. *gouldi* symbiont phylotype B (one of the most common [27, 28]). This investigation is of particular interest given the extracellular location and facultative nature of thyasirid symbionts, and provides a contrast to genomic studies of intracellular (and often obligate) bivalve chemosymbionts. Based on the metagenomic data collected, we characterize important metabolic cycles, including carbon fixation and sulfur oxidation, and identify genomic characteristics that allow us to infer the mode of symbiont transmission and support the evidence for a free-living state in thyasirid bacterial symbionts. By identifying the genes for metabolic pathways in symbiont genomes, we lay the groundwork for future transcription and protein studies.

## Results and discussion

### Overview of metagenome

Our analysis recovered a large number of contigs (12,504, with an N_50_ of 1870) that we consider to represent the symbiont population’s metagenome; these contigs could not be assembled into a single genome. A high level of sequence redundancy, with sequences of 54% of contigs being significantly similar to at least one other contig (Blast Evalue < 10^− 10^; Additional file 1) suggests many cases of slight sequence divergence across the symbiont (population) metagenome. The contigs from our metagenome show highly similar trinucleotide and tetranucleotide frequency distributions (i.e. tight clusters in Principal Components Analyses; Additional file 1), supporting our interpretations that: 1) the sequences we retained as belonging to symbionts (i.e., the metagenome) come from highly similar bacteria; and 2) the metagenome contained few contaminants, as expected given the very high abundance of morphologically similar symbionts observed on *Thyasira* cf. *gouldi* gills using transmission electron microscopy [20]. This metagenome is most likely a combination of multiple bacterial genomes recovered from a population of symbionts that share the same 16S rRNA gene sequence (only one complete 16S rRNA gene sequence was found, for phylotype B), but with divergence in gene arrangement and gene sequence across the population. Most likely, the genomes across the symbiont population were highly similar overall, but contained regions of sufficient variation to prevent assembly of contigs into a single genome, accounting for our inability to quantify genomic size. The heterogeneous (non-clonal) nature of thyasirid symbiont populations was suggested previously [28]. Hereafter, we describe features of this metagenome, considering that it represents a similar, yet divergent population of *T*. cf. *gouldi* symbionts sharing the same 16S rRNA phylotype B gene sequence.

The average read depth coverage is 33, with 50% of bases having a depth coverage of 14 or higher. Bases within areas identified as genes had an average read depth coverage of 31 with 50% having a depth coverage of 20 or higher. The GC content is 42 ± 7%, lower than the symbionts of *Solemya velum* (51%) and *Riftia pachyptila* (58%) and higher than the symbionts of *Calyptogena magnifica* (34%) and *Calyptogena okutanii* (32%); it is similar to the GC content of the free-living bacteria *Thiomicrospira crunogena* (43%) [17]. In total, 20,843 putative open reading frames (ORFs) were detected in the contigs: this number likely includes variants of the same genes present within different genomes across the metagenome. Using the MG-RAST workflow, possible functions were assigned to 3339 of the identified ORFs, allowing us to infer some of the metabolic capabilities of the *T.* cf. *gouldi* symbionts. Assigned gene functions were grouped into subsystems (curated collections of genes associated with specific metabolic pathways, biological processes, structural complexes or protein families [31]) using the MG-RAST workflow (Table 1).

**Table 1:** The number of genes assigned to functional subsystems using MG-RAST

Subsystem Functions	Number of genes assigned
Amino Acids and Derivatives	211
Carbohydrates	177
Miscellaneous	151
DNA Metabolism	136
Protein Metabolism	148
Cell Wall and Capsule	113
Cofactors, Vitamins, Prosthetic Groups, Pigments	109
RNA Metabolism	107
Regulation and Cell signaling	106
Respiration	102
Membrane Transport	90
Stress Response	66
Virulence, Disease and Defense	61
Nitrogen Metabolism	54
Phages, Prophages, Transposable elements, Plasmids	42
Fatty Acids, Lipids, and Isoprenoids	40
Motility and Chemotaxis	38
Nucleosides and Nucleotides	38
Sulfur Metabolism	30
Phosphorus Metabolism	25
Cell Division and Cell Cycle	25
Metabolism of Aromatic Compounds	22
Other	382

### Genomic support of environmental transmission

Some general characteristics of the metagenome are consistent with *T.* cf. *gouldi* symbionts having a free-living stage: there is no sign of genomic reduction (genes present were assigned to a broad range of functions, with no essential category appearing to be missing) and mobile elements are present [32]. While this is not conclusive evidence of environmental transmission, they are uncommon in co-evolved vertically transmitted symbionts [32]; other evidence, such as the identification of the symbiont 16S rRNA gene in surrounding sediments [30], supports a free-living capability.

Four different mobile elements (Tn10 transposons, ISPsy4, IS200, and a MULE transposase domain possibly from IS256) were identified using the MG-RAST website and PROKKA (see Methods), although the exact number of copies was unclear because of the fragmented nature of metagenome. Phage genes belonging to the order Caudovirales, similar to T5 phages, were also identified. Genes for phage tail, capsid, and recombinase were identified, although their exact number was again indiscernible. There was no apparent loss of genes for DNA repair in the *T.* cf. *gouldi* symbiont metagenome (Table 2), in contrast to the reduced genome of the vesicomyid symbionts which lack *recA* for genetic recombination and *mutY* for DNA repair [33]. The loss of genes for DNA repair has been observed in vertically transmitted symbionts, contributing to GC bias and the presence of many pseudogenes [32, 33].

**Table 2:** Putative gene functions associated with the Calvin-Benson-Bassham Cycle

Putative gene function	Number of copies in the metagenome	Percent identity	Alignment length (bp)
Fructose-bisphosphate aldolase class I	1	78.93	337
Fructose-bisphosphate aldolase class II	1	75.42	354
NAD-dependent glyceraldehyde-3-phosphate dehydrogenase	2	80.39	225.5
NADPH-dependent glyceraldehyde-3-phosphate dehydrogenase	1	83.54	328
Phosphoglycerate kinase	1	71.58	285
Phosphoribulokinase	2	80.21	96
Ribose 5-phosphate isomerase A	4	73.16	71.75
Ribulose bisphosphate carboxylase	2	83.3	180
Ribulose-phosphate 3-epimerase	1	84.68	222
Transketolase	1	71.61	384
Transketolase, C-terminal section	2	62.81	112.5
Transketolase, N-terminal section	1	61.73	81
Triosephosphate isomerase	1	67.07	167

The metagenomic data showed genes associated with flagellar assembly and function (*flaG*, *flgA*, *B*, *C*, *E*, *F*, *G*, *H*, *I*, *J*, *K*, *L*, *flhA*, *B*, *F*, *fliD*, *E*, *G*, *H*, *K*, *L*, *M*, *N*, *P*, *Q*, *S*, *T*, *U*, *W*, *mcpB*, *mcpS*, *motB*, *motD*, *pctC*, *pomA*, *swrC*, *tar*, *ycgR*, and an undefined flagellar motor protein). Also identified were the Che genes (*cheA*, *B*, *R*, *V*, *W*, *Y*, and *Z*), which can detect chemical conditions in the environment, and interact with the flagellar motor to help the bacteria move to suitable areas within the environment [34]. These genes are essential to locate and move to the micro-oxic, reduced sulfur rich areas of the sediment this bacterium needs for sulfur oxidation. An aerotaxis gene (similar to *aer*) was also identified, likely allowing the bacteria to locate the micro-oxic areas where sulfur oxidation is most efficiently carried out [35]. When associated with a host, reduced sulfur is made accessible to symbionts by the sulfur mining behavior of the clam; however, bacteria in the free-living population most likely require key genes for substrate location and motility [30]. Like the environmentally transferred *R. pachyptila* symbiont*,* the *T.* cf. *gouldi* symbiont has a full complement of flagellar genes, as well as an array of chemotaxis genes [16]. Surprisingly, no magnetotaxis genes were identified by our metagenomic analysis, although magnetosome particles were identified in the symbionts of *T*. cf. *gouldi* [30]. Magnetosome genes are likely present in the genome, but either were not sequenced or could not be identified by the pipeline employed here. A directed BLAST search using known magnetosome genes *mam*, *man*, *mms*, and *mad* did not yield any results. Interestingly, these genes have not yet been identified in magnetotactic gammaproteobacteria; this taxon may simply lack comparable genetic information.

**Fig. 1:**
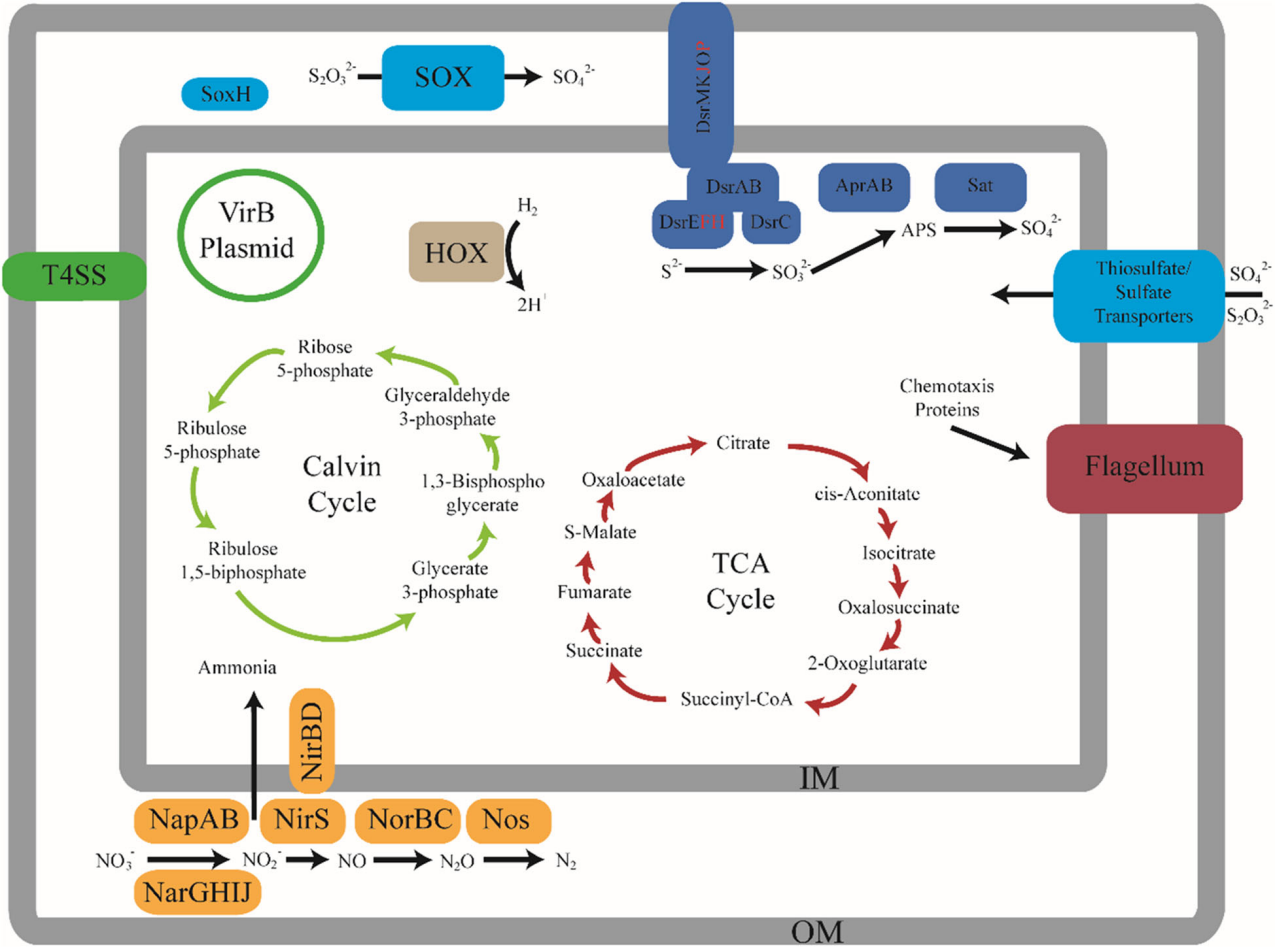
Schematic representation of inferred metabolic capabilities of the *Thyasira* cf. *gouldi* symbiont. The major metabolic cycles discussed in this paper are highlighted within the figure. The inner membrane is designated by IM and the outer membrane by OM. Genes that were not identified within either annotation pipeline are designated by a red letter

A schematic representation of inferred metabolic capabilities of the *T.* cf. *gouldi* symbiont is presented in Fig. 1. The symbiont may not be restricted to thiotrophy and may be able to use alternative metabolic pathways when reduced sulfur is not available. In culturing experiments, the sulfur oxidizing bacterium *Sedimenticola thiotaurini* SIP-G1 is unable to fix carbon in oxic conditions, where it must instead rely on heterotrophy [36]. A previous phylogenetic study [28] placed the *T.* cf. *gouldi* symbiont near *S. thiotaurini* SIP-G1. Based on this phylogenetic placement and the genes identified by this study, the *T.* cf. *gouldi* symbiont may have similar metabolic capabilities; however, without culturing the bacteria we cannot validate this theory.

### Amino acid and cofactor synthesis

Symbionts commonly retain genes important for amino acid, vitamin, and cofactor production because the host selects for bacteria that provide the nutrients it requires [15]. Many putative gene functions of the *T.* cf. *gouldi* symbiont are involved in amino acid transport and metabolism (229 assignments; Table 1), while cofactor transport and metabolism are also frequently identified (112 times; Table 1). These functions are also present in free-living bacteria, so while important for the symbiosis, they are also presumably essential to the bacteria outside the host.

### Thioautotrophy

In the *T.* cf. *gouldi* symbiont, the metabolic cycles for carbon fixation and sulfur oxidation are of particular interest. Several genes for the sox and dsr pathways (likely an oxidative dsr pathway) are present (see below), and the symbiont may conduct sulfur oxidation through both these pathways. Both these cycles have been found to function simultaneously in bivalve and vestimentiferan chemosymbionts [37–39]. Sulfur compounds within the benthic sediment are patchy, and therefore being able to utilize different forms would increase the habitat range for these bacteria and their bivalve hosts.

*SoxA*, *X*, *Y* and *Z*, which are found in a cluster in the genome of vesicomyid symbionts [37] and form a multi-enzyme system that can oxidize various forms of reduced sulfur (sulfide, thiosulfate, elemental sulfur and sulfite) to sulfate [37, 40] were found in the *T.* cf. *gouldi* metagenome. We found no evidence for soxCD, which is found in some sulfur-oxidizing bacteria but is lacking in others (including in the *Calyptogena* symbiont [37]). The lack of soxCD can manifest itself by the presence of bacterial sulfur globules, which appear as white inclusions in transmission electron micrographs of *T*. cf. *gouldi* symbionts, due to sulfur removal during processing (e.g. Fig. 2B of [20]). The *T*. cf. *gouldi* symbiont metagenome included *soxH*, a peripheral, thiosulfate inducible sox gene that is located in the periplasm but is not essential for growth on thiosulfate and has an unknown function [41]. We also identified *cysA*, shown to import both sulfate and thiosulfate from the environment [42]. Adenylylsulfate reductase was found, and its activity was previously detected in thyasirid symbionts [23, 25].

Many of the genes in the dsr cycle were found, with the *dsrA*, *B*, and *C* proteins as well as the peripheral *dsrE* suggesting that the pathway is running in an oxidative direction [43]. These genes as well as *dsrK*, *M*, *R*, *S* are present in the symbiont genome. The vestimentiferan symbionts discussed by Li et al. did not contain the *dsrMKJOP* membrane bound protein complex [39]. An oxidative dsr pathway is present in many well-studied symbionts, including those associated with multiple *Calyptogena* species, *R. pachyptila*, and *Crysomallon squamiferum* [16, 37, 44].

Thirteen putative functions associated with the Calvin-Benson-Bassham Cycle were discovered in the *T.* cf. *gouldi* symbiont metagenome (Table 2). The Calvin-Benson-Bassham Cycle in the *T*. cf. *gouldi* symbiont utilizes a type II RuBisCO enzyme [20, 28]. Chemosymbionts of bivalves often have a reversible pyrophosphate-dependent phosphofructokinase in place of the sedoheptulose-1,7-bisphosphatase that this enzyme replaces, and the fructose 1,6 bisphosphatase genes, which are employed in a reverse TCA cycle [17, 38]. However, we were unable to identify any of these three genes in our analysis, but did find ribose 5-phosphate isomerase, which is used in the typical Calvin-Benson-Bassham pathway but is missing in the symbionts of *Calyptogena magnifica* and *R. pachyptila* [15, 16]. It is not clear if the thyasirid symbiont has a traditional Calvin-Benson-Bassham cycle, or if the modifications common in other sulfur oxidizing symbionts are also present in this symbiont [17, 38].

### Hydrogen oxidation

The symbiont also appears capable of hydrogen oxidation using the NAD^+^-reducing hydrogenase *hoxHYUF*, and a second set of closely related genes identified as the alpha, beta, delta, and gamma subunits of *hoxS*. The enzyme produced by these complexes is bi-directional. It has been described previously in the symbiont of some vestimentiferan worms [39, 45] as well as free-living *Sedimenticola selenatireducens* [46].

### Heterotrophy

Genes associated with the tricarboxylic acid (TCA) cycle were also identified in the *T.* cf. *gouldi* symbiont (Table 3). Interestingly, the TCA cycle in this symbiont does not appear to use the oxoglutarate shunt and contains both the α ketoglutarate dehydrogenase and citrate synthase enzymes which are commonly lost in chemosymbiotic bacteria and cause the loss of heterotrophic abilities [17]. All genes for a functional TCA cycle have been found in the chemosymbiont of *Solemya velum*, which may occur outside of its host [17]. The genome of the *R. pachyptila* symbiont also encodes a complete TCA cycle and contains evidence for response to carbon compounds in the environment, suggesting that it can survive heterotrophically outside the host [16]. Sugar phosphotransferase systems (PTS) were also identified in our dataset. These systems can import sugars from the environment, increasing the evidence for some heterotrophic ability. Sugar PTS were identified for fructose, mannose, galactose, and sucrose, suggesting that these substrates can be acquired from the environment, supplementing carbon fixation. In pure culture, the sediment bacterium *S. thiotaurini* SIP-G1 is unable to grow on sulfur oxidation alone and must be provided with heterotrophic nutrients [36]. A similar system may exist within the *T.* cf. *gouldi* symbiont, with heterotrophic growth occurring when environmental conditions are unfavorable for carbon fixation. The ability to utilise multiple carbon sources would be very beneficial during a free-living stage, especially in seasonally variable environments where sulfur compounds can be scarce, and availability of organic matter fluctuates.

**Table 3:** Putative gene functions involved in a complete TCA cycle identified by MG-RAST

Putative gene function	Number of copies	Percent identity	Alignment length (bp)
2-oxoglutarate dehydrogenase E1 component	1	70.33	209
Aconitate hydratase	5	65.53	109
Aconitate hydratase 2	1	72.11	190
Citrate synthase (si)	1	66.97	109
Dihydrolipoamide dehydrogenase	2	73.13	89
Dihydrolipoamide dehydrogenase of pyruvate dehydrogenase complex	1	75.83	211
Dihydrolipoamide succinyltransferase component (E2) of 2-oxoglutarate dehydrogenase complex	2	75	138.5
Fumarate hydratase class I, aerobic	2	88.05	163
Isocitrate dehydrogenase [NADP]	3	69.21	265.33
Malate dehydrogenase	2	63.59	209
Succinate dehydrogenase flavoprotein subunit	4	77.19	137.5
Succinate dehydrogenase iron-sulfur protein	1	70.83	144
Succinyl-CoA ligase [ADP-forming] alpha chain	3	81.71	185
Succinyl-CoA ligase [ADP-forming] beta chain	4	72.13	178

### Anaerobic respiration

The *T.* cf. *gouldi* symbiont appears to be capable of performing denitrification, as the genes for the nar and nos pathways are present in the metagenome. Denitrification is the process that reduces potentially harmful nitrogen compounds (nitrates, nitrites, and nitric oxide) into harmless, inert N_2_ through anaerobic respiration. Denitrification may provide multiple advantages to both host and symbiont, in addition to allowing bacterial ATP synthesis. First, by reducing harmful nitrogenous compounds, the bacteria may protect their host from toxic effects. Second, by decreasing the symbiont’s oxygen requirements, there is less competition with the host for this limited resource in the thyasirid’s endobenthic environment. Third, the pathways could allow the bacteria to respire anaerobically in anoxic sediments, and therefore broaden the organism’s free-living niche. Notably, the closely related free-living bacterium *S. thiotaurini* SIP-G1 from salt marsh sediments is capable of anaerobic respiration using nitrate and nitrite, but can also grow under hypoxic conditions [36]. Dissimilatory nitrate respiration genes have also been identified in the symbionts of *Vesicomyosocius okutanii*, *Bathymodiolus thermophilus*, and a number of vestimentiferan tubeworms [16, 39, 47, 48].

Recent work has shown some sulfur-oxidizing chemosymbionts can also fix atmospheric nitrogen into bioavailable forms [18, 49], however, we did not find any evidence of N fixation genes in the *T.* cf. *gouldi* symbiont. The closely-related free-living bacterium *S. thiotaurini* SIP-G1 does however have a complete nitrogen fixation pathway [36].

## Conclusions

The metagenomic data collected corroborates previous data suggesting a facultative relationship between *T.* cf. *gouldi* and its symbionts, with the host clams being inoculated from the environment [28, 30]. The timing of this inoculation during the host’s lifespan is still unclear and further research is needed to determine when the host is competent for symbiont uptake. The symbiont population is a collection of closely related individuals, although the population is not clonal and some variation is present. There is no evidence of genome reduction in these symbionts, and the genomic data supports evidence of an environmental (non-symbiotic) habitat. In particular, the presence of a functional flagellum and chemosensory abilities supports the presence of a free-living population, as reported previously [30].

The metabolic capabilities of the symbionts are comparable to previously described sulfur oxidizing bacteria. The symbionts utilize multiple pathways for sulfur oxidation, both sox and dsr, and the Calvin-Benson-Bassham Cycle for carbon fixation. The denitrification pathway that is also present would allow for ATP generation in anaerobic areas; when outside the host, sulfides are predominantly found in micro-oxic areas. Unlike many obligate symbionts, the thyasirid symbiont appears to have a functional TCA cycle and sugar importers allowing it to be heterotrophic. The bacteria may utilize autotrophy or heterotrophy under different conditions, like *S. thiotaurini* SIP-G1 [36].

Further research into the thyasirid symbiont genome may be beneficial in tracking the changes required for life as a bivalve symbiont, and experimental studies could reveal whether symbionts are capable of reverting to a non-symbiotic state after they have become associated with their host. The *T.* cf. *gouldi* symbiosis provides a unique opportunity to investigate how symbioses evolve as this appears to be a relatively less derived and interdependent relationship compared to other bivalve symbioses which are intracellular. More research into the metabolic capabilities of the symbiont and how they interact with the host would provide insights into how this relationship has evolved, and the mechanisms that allow it to be maintained. Comparing the different symbiont phylotypes capable of associating with a single host species would also improve our understanding of this relationship and of the potential benefits of flexible host-symbiont pairings. Future studies would benefit from the application of more powerful sequencing technologies, such as mate-paired sequencing or single-cell genomic sequencing, which would facilitate genomic comparison with other symbionts and free living bacteria.

## Methods

### Sample collection and sequencing

Sediment was collected in August 2010 using a Petersen grab from Neddy’s Harbour, in the fjord of Bonne Bay, Newfoundland, Canada (49°31′21.44″N, 57°52′11.07″W), at a depth of roughly 15 m. Sediment was wet sieved using a 1 mm mesh and specimens of *T.* cf. *gouldi* were collected and transported to Memorial University, St. John’s, Newfoundland. Host individuals were collected during times of high symbiont abundance [50], and visually inspected for indication of high symbiont population. To reduce environmental contamination of bacteria not contained within the mucus between gill filaments, surface of the gill was rinsed before DNA extraction. Total DNA was extracted from the gills of a single individual (host OTU 1 [20]) using a Qiagen Blood and Tissue Kit and stored at − 20 °C in the elution buffer provided. Before sequencing, total DNA was transferred to nuclease free water. An Ion Torrent Fragmentation Kit was used and fragments of approximately 200 bp were selected using gel size selection and extraction (Qiagen Gel Extraction Kit), purified (Qiagen DNA Purification Kit) following manufacturer’s instructions, and concentrations assessed with an Agilent Bioanalyser. Sequencing was conducted on an Ion Torrent PGM Sequencer following the manufacturer’s protocols (V2.2). A 316 chip was used for sequencing. Due to poor load rates, two sequencing runs were conducted and the data were combined before further processing.

### Assembly and annotation

Reads were quality checked and trimmed using FastQC, FastQ Groomer, FastQ Quality Trimmer, and Filter FastQ on the Galaxy Website (usegalaxy.org) and FastQC software [51, 52]; any reads less than 50 bp long were removed at this stage. A quality score of 20 was used, allowing one base below the cutoff score within the read, and trimming was conducted on both ends. Filtered reads were assembled into contigs and then binned using MEGAN5 [53], which used BLAST to compare each contig to the nr database. Reads from all contigs identified as “bacteria”, “not assigned”, “unclassified” or “no hits” were identified and retained for reassembly and subsequent analysis; the combination of these reads was deemed to represent the symbiont metagenome.

Assembly of the binned data (i.e., deemed to represent the symbiont metagenome) was conducted using SPAdes [54]. Ion Torrent specific settings with kmers 27, 35, 55 and 77 were used. Annotation was run using the MG-RAST website (http://metagenomics.anl.gov/) [55], the RefSeq, KOG, and Subsystems databases were used with the e-value cut-off set at 5, % identity 60, min length 15, and min abundance 1. A secondary annotation was conducted using PROKKA with the default settings provided [56].

Principal component analysis (PCA) was performed on the trinucleotide and tetranucleotide frequency distribution of the read sequences. All 5.5 million reads were mapped back to the contigs assembled by SPAdes using BWA-MEM [57] version 0.7.17 with default settings. The 25% of reads that did not align to the contigs were identified using SAMtools [58]. Trinucleotide and tetranucleotide frequency distribution of the read sequences were obtained using the R function oligonucleotideFrequency available in the Biostrings package [59]. PCA was carried out using the R function prcomp with scale and center set to true. PCA results were visualized using the R function autoplot available in the ggfortify package [60]. Reads were coloured based on whether they were or not aligned to the contigs. To investigate whether the contigs identified by MEGAN5 as “eukaryote” have a distinct trinucleotide frequency distribution from the contigs deemed belonging to the bacterial metagenome, PCA as described above was performed on the trinucleotide frequency distribution of the contigs sequences.

## Supplementary information

**Additional file 1: Figure S1.** Total number of contigs (log2 scale) with a significant (Evalue <10e-10) local sequence similarity with *N* other contigs. *N* is indicated on the x-axis. Approximately 55% of the 12,504 contigs assembled have significant local sequence similarity with at least one other contig. **Figure S2.** PCA of the trinucleotide (left) and tetranucleotide (right) frequency distribution of the raw reads. Aligned “Yes” indicates the reads that were aligned back to the contigs deemed to belong to the symbiont metagenome. On each axis label the number between brackets is the percentage of variance explained by the corresponding principal component. Ellipses are 95% data ellipses assuming a multivariate Gaussian distribution. Ellipses have the mean vector as their center and cover 95% of the corresponding data points. The reads aligned to the metagenome have low variance in terms of their tri(tetra)nucleotide frequency. **Figure S3.** PCA of the trinucleotide frequency distribution of the putative symbiont contigs (metagenome) and eukaryotic contigs as classified by MEGAN5. On each axis label the number between brackets is the percentage of variance explained by the corresponding principal component. Ellipses are 95% data ellipses assuming a multivariate Gaussian distribution. Ellipses have the mean vector as their center and cover 95% of the corresponding data points. The symbiont metagenome have low variance in terms of their trinucleotide frequency distribution and are clearly more homogenous than the eukaryotic contigs.

## Data Availability

Sequence reads can be found on the SRA database under sample SRS1569030, sequencing runs SRR3928943 and SRR3928944. Assembled contigs were uploaded to GenBank under BioProject PRJNA327811, Biosample SAMN05358035, accession numbers MOXF01000001.1-MOXF01012504.1.

## Abbreviations

ATP: Adenosine triphosphate
C: Carbon
dsr: Dissimilatory sulfate reduction
IM: Inner membrane
N: Nitrogen
NAD: Nicotinamide adenine dinucleotide
NADPH: Dihydronicotinamide-adenine dinucleotide phosphate
nir: Nitrate reduction
OM: Outer membrane
ORF: Open reading frame
OTU: Operational taxonomic unit
PCA: Principal components analysis
PCR: Polymerase chain reaction
PTS: Phosphotransferase system
rRNA: Ribosomal RNA
RuBisCO: Ribulose-1,5-bisphosphate carboxylase/oxygenase
sox: Sulfur oxidation
TCA: Tricarboxylic acid
