# Supplementary material for: Metagenomic analysis suggests broad metabolic potential in extracellular symbionts of the bivalve Thyasira cf. gouldi
Source: Anim Microbiome. 2020 Mar 5;2:7. doi: 10.1186/s42523-020-00025-9 (PMC7807488; doi:10.1186/s42523-020-00025-9)
Supplement: Supplementary file 1 — Additional file 1: Figure S1. Total number of contigs (log2 scale) with a significant (Evalue <10e-10) local sequence similarity with N other contigs. N is indicated on the x-axis. Approximately 55% of the 12,504 contigs assembled have significant local sequence similarity with at least one other contig. Figure S2. PCA of the trinucleotide (left) and tetranucleotide (right) frequency distribution of the raw reads. Aligned “Yes” indicates the reads that were aligned back to the contigs deemed to belong to the symbiont metagenome. On each axis label the number between brackets is the percentage of variance explained by the corresponding principal component. Ellipses are 95% data ellipses assuming a multivariate Gaussian distribution. Ellipses have the mean vector as their center and cover 95% of the corresponding data points. The reads aligned to the metagenome have low variance in terms of their tri(tetra)nucleotide frequency. Figure S3. PCA of the trinucleotide frequency distribution of the putative symbiont contigs (metagenome) and eukaryotic contigs as classified by MEGAN5. On each axis label the number between brackets is the percentage of variance explained by the corresponding principal component. Ellipses are 95% data ellipses assuming a multivariate Gaussian distribution. Ellipses have the mean vector as their center and cover 95% of the corresponding data points. The symbiont metagenome have low variance in terms of their trinucleotide frequency distribution and are clearly more homogenous than the eukaryotic contigs. [file 42523_2020_25_MOESM1_ESM.pdf]

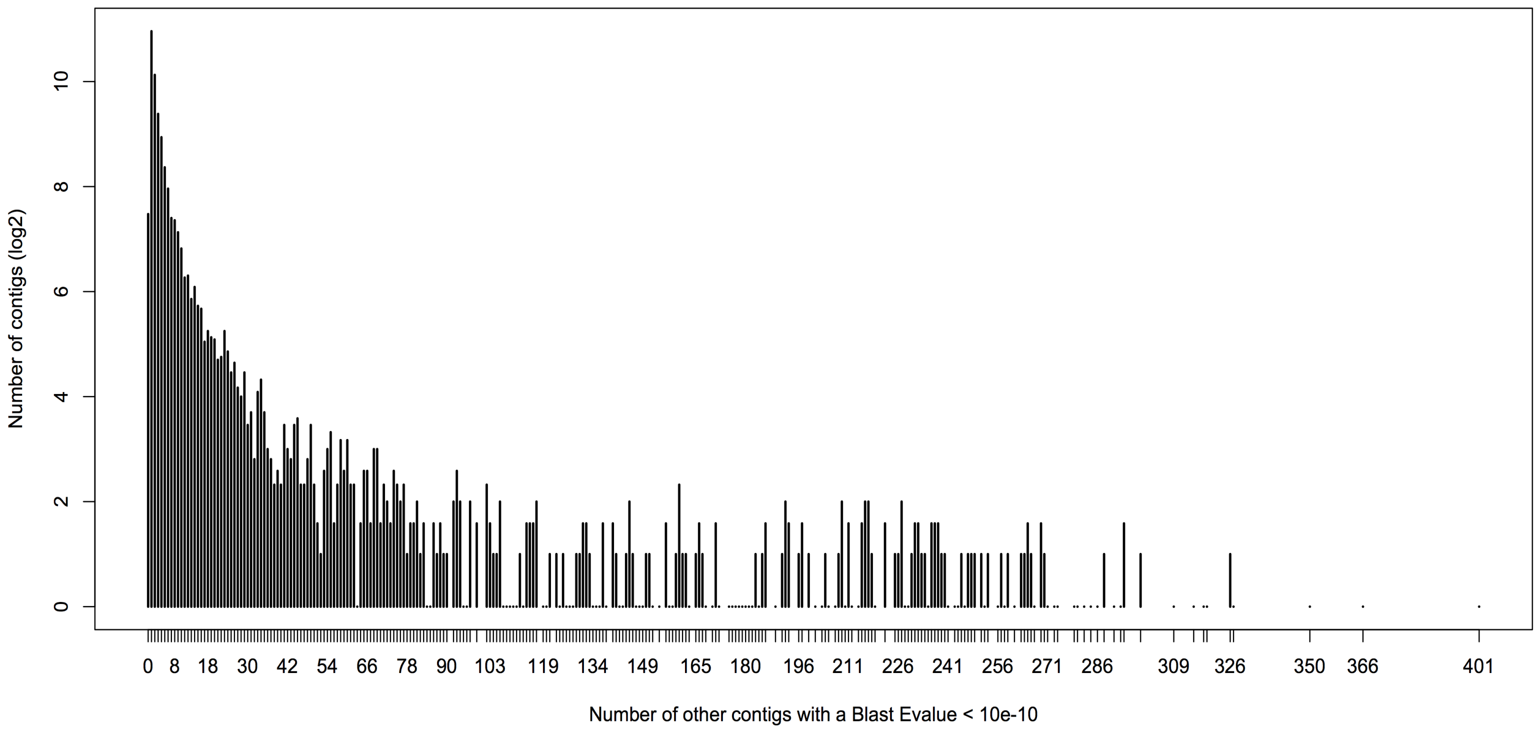


Supplementary Fig 1. Total number of contigs (log2 scale) with a significant (Evalue < 10e-10) local sequence similarity with *N* other contigs. *N* is indicated on the x-axis. Approximately 55% of the 12,504 contigs assembled have significant local sequence similarity with at least one other contig.

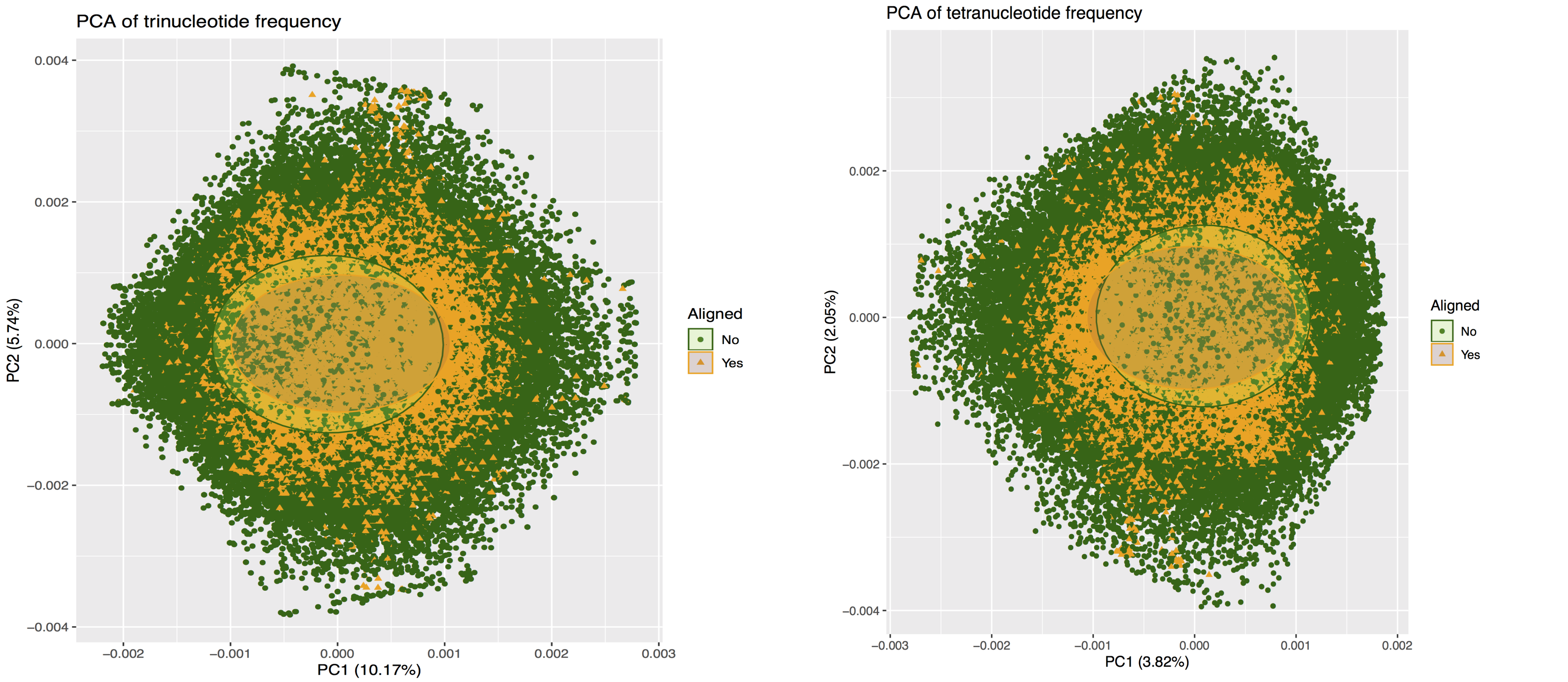


Supplementary Figure 2. PCA of the trinucleotide (left) and tetranucleotide (right) frequency distribution of the raw reads. Aligned "Yes" indicates the reads that were aligned back to the contigs deemed to belong to the symbiont metagenome. On each axis label the number between brackets is the percentage of variance explained by the corresponding principal component. Ellipses are 95% data ellipses assuming a multivariate Gaussian distribution. Ellipses have the mean vector as their center and cover 95% of the corresponding data points. The reads aligned to the metagenome have low variance in terms of their tri(tetra)nucleotide frequency.

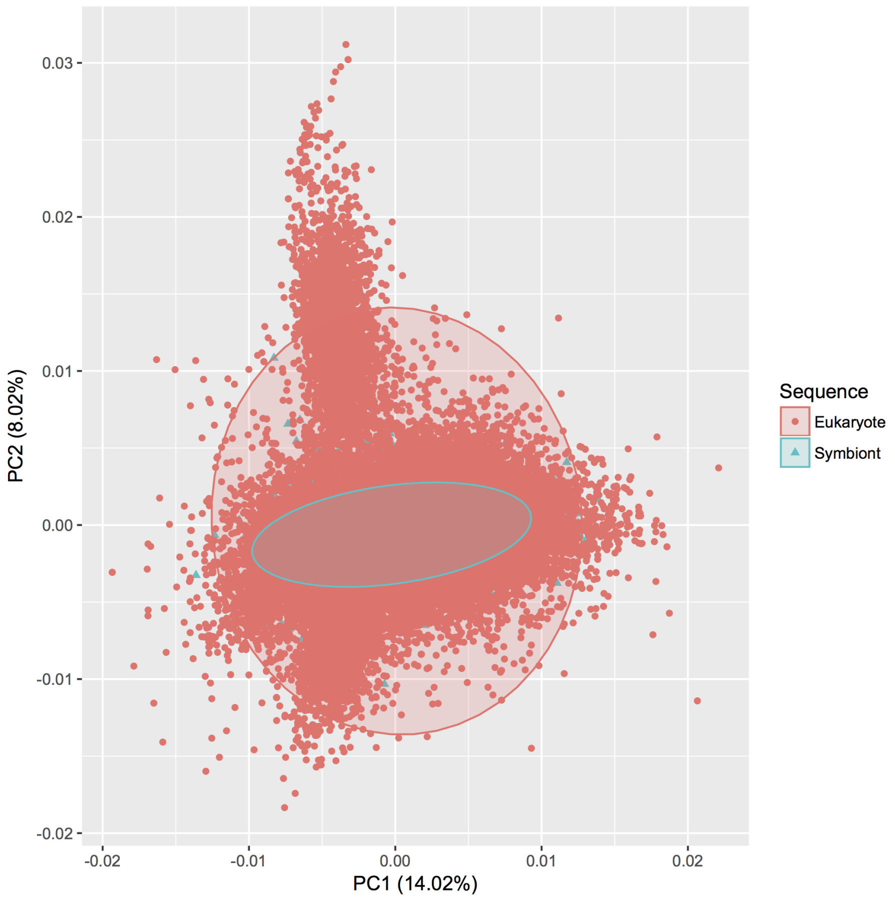


Supplementary Figure 3. PCA of the trinucleotide frequency distribution of the putative symbiont contigs (metagenome) and eukaryotic contigs as classified by MEGAN5.  On each axis label the number between brackets is the percentage of variance explained by the corresponding principal component. Ellipses are 95% data ellipses assuming a multivariate Gaussian distribution. Ellipses have the mean vector as their center and cover 95% of the corresponding data points. The symbiont metagenome have low variance in terms of their trinucleotide frequency distribution and are clearly more homogenous than the eukaryotic contigs.
